# Potent programmable antiviral against dengue virus in primary human cells by Cas13b RNP with short spacer and delivery by VLP

**DOI:** 10.1016/j.omtm.2021.04.014

**Published:** 2021-05-01

**Authors:** Ekapot Singsuksawat, Suppachoke Onnome, Pratsaneeyaporn Posiri, Amporn Suphatrakul, Nittaya Srisuk, Rapirat Nantachokchawapan, Hansa Praneechit, Chutimon Sae-kow, Pala Chidpratum, Khanit Sa-ngiamsuntorn, Suradej Hongeng, Panisadee Avirutnan, Thaneeya Duangchinda, Bunpote Siridechadilok

**Affiliations:** 1 National Center for Genetic Engineering and Biotechnology, Klong Luang, Pathumthani 12120, Thailand; 2 Division of Dengue Hemorrhagic Fever Research, Faculty of Medicine Siriraj Hospital, Mahidol University, Bangkok 10700, Thailand; 3 Department of Pediatrics, Faculty of Medicine, Ramathibodi Hospital, Mahidol University, Bangkok 10400, Thailand; 4 Siriraj Center of Research Excellence in Dengue and Emerging Pathogens, Faculty of Medicine Siriraj Hospital, Mahidol University, Bangkok 10700, Thailand; 5 Graduate Program in Immunology, Department of Immunology, Faculty of Medicine Siriraj Hospital, Mahidol University, Bangkok 10700, Thailand; 6 Department of Biochemistry, Faculty of Pharmacy, Mahidol University, Bangkok 10400, Thailand

**Keywords:** flavivirus, RNA virus, CRISPR-Cas13, delivery, and virus-like particle, VLP, dengue, zika

## Abstract

With sequencing as a standard frontline protocol to identify emerging viruses such Zika virus and severe acute respiratory syndrome coronavirus 2 (SARS-CoV-2), direct utilization of sequence data to program antivirals against the viruses could accelerate drug development to treat their infections. CRISPR-Cas effectors are promising candidates that could be programmed to inactivate viral genetic material based on sequence data, but several challenges such as delivery and design of effective CRISPR RNA (crRNA) need to be addressed to realize practical use. Here, we showed that virus-like particle (VLP) could deliver PspCas13b-crRNA ribonucleoprotein (RNP) in nanomolar range to efficiently suppress dengue virus infection in primary human target cells. Shortening spacer length could significantly enhance RNA-targeting efficiency of PspCas13b in mammalian cells compared to the natural length of 30 nucleotides without compromising multiplex targeting by a crRNA array. Our results demonstrate the potentials of applying PspCas13b RNP to suppress RNA virus infection, with implications in targeting host RNA as well.

## Graphical abstract

**Figure:**
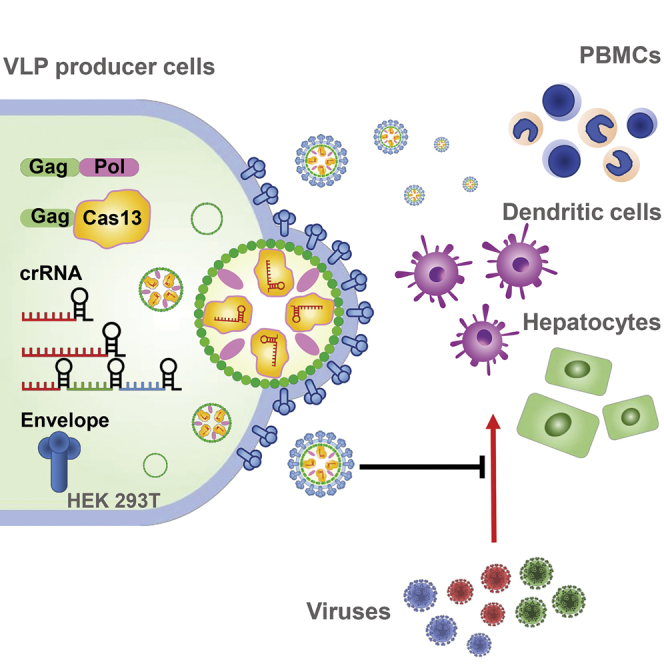

## Teaser

We construct a virus-like particle that can efficiently deliver Cas13b-crRNA ribonucleoprotein complex into primary human cells to inhibit dengue virus infection. In addition, short spacer length of crRNA (22–26 nucleotides) can enhance Cas13b knockdown activity in mammalian cells without interfering with crRNA processing and enabling multi-virus targeting.

## Introduction

Among the emerging pathogenic human viruses, the majority are RNA viruses. In recent years, large outbreaks of Zika viruses, Ebola viruses, and, currently, severe acute respiratory syndrome coronavirus 2 (SARS-CoV-2) have prompted the World Health Organization (WHO) to declare global health emergencies. Vaccine and antiviral drugs are key arsenals to combat these viruses. While vaccine development can now take full advantage of virus sequences to promptly generate vaccine leads, current antiviral drug development does not have this capability. CRISPR-Cas13, bacterial proteins that can be programmed to target specific RNA with crRNA, are promising molecules for developing programmable antivirals against RNA viruses. Programmability of CRISPR-Cas13 provides the flexibility to target a broad range of RNA viruses.^1^^,^^2^

CRISPR-Cas13 belongs to class II, type VI CRISPR-Cas system with a single effector nuclease that targets RNA. The presence of higher eukaryotes and prokaryotes nucleotide (nt)-binding domains (HEPN-1 and HEPN-2) is a defining feature of Cas13. Cas13 utilizes crRNA to target specific RNA. crRNA is a small noncoding RNA with a linear spacer region that base pairs with target RNA and the direct-repeat (DR) region that forms a hairpin structure and binds to Cas13 protein. Cas13 and crRNA form a ribonucleoprotein (RNP) complex to target specific RNA. Once Cas13 RNP binds to target RNA, the nuclease activity on Cas13 is activated.^3^ The nuclease activity can cleave both the bound RNA (*cis* cleavage) and other RNA molecules nearby (*trans* cleavage).^4^^,^^5^ Three CRISPR-Cas13 subtypes (CRISPR-Cas13a, 13b, and 13d) have been shown to be capable of knocking down genes in a variety of eukaryotic cells with high specificity.^6^, ^7^, ^8^

CRISPR-Cas13 has been shown to reduce infections of several human RNA viruses in cell lines.^1^^,^^9^, ^10^, ^11^, ^12^, ^13^ These studies show efficient viral RNA targeting by CRISPR-Cas13. However, several challenges remain to translate it into practical use. Transient delivery of Cas13b-crRNA into target cells is desirable for treating an acute viral infection to avoid potential toxicity of long-term expression of Cas13 in the cells and to minimize immune response against Cas13 in repeated dosing. The challenges in designing potent crRNAs with broad targeting ability are being addressed.^9^^,^^14^ Recently, a demonstration of antiviral activities of CRISPR-Cas13 against influenza and SARS-CoV-2 was achieved in mice and hamsters, respectively. Cas13a was transiently delivered as mRNA along with crRNA in the form of lipid nanoparticles (LNPs), providing a key proof of concept for *in vivo* application.^15^ The study focused on respiratory viruses that could be treated with local administration such as nebulization. Development of systematic delivery vehicles to target multiple tissues that are targets of several RNA viruses is still needed. Here, we showed that transient delivery of Cas13b RNP in nanomolar range by virus-like particle (VLP) could efficiently suppress dengue virus (DENV) infection in several primary human cells. In contrast to previous studies *in vitro* and in bacteria, we found that shortening spacer length of crRNA in the range of 18–26 nts could enhance knockdown activity by Cas13b in mammalian cells and did not compromise crRNA processing and multiplex targeting capability.

## Results

### Characterization of CRISPR-Cas13b RNA targeting activity against DENV and ZIKV

To characterize the antiviral activity of CRISPR-Cas13 against flavivirus, we first established a stable BHK-21 clone (BHK-21-Cas13b) that could be induced with doxycycline to express PspCas13b using lentivirus (Figure S1A). Type I-interferon defective BHK-21 cell line was chosen as the host cell for its ability to support high level of DENV and Zika virus (ZIKV) replication, providing a robust platform to evaluate viral suppression activity. PspCas13b was chosen for its strong knockdown activity when localized in the cytoplasm,^6^ the location of flavivirus RNA replication. Inducible expression system appeared to provide the stability of Cas13b cassette in BHK-21 as we were not able to maintain PspCas13b expression under a constitutive promoter (EF1a) over several cell passaging (data not shown). To test virus suppression, we utilized both fluorescent reporter viruses, which provided virus-encoded fluorescent readout of virus replication,^16^^,^^17^ and natural strains DENV2-16681 and ZIKV-SV0010.^18^ We found that cytoplasmic PspCas13b could suppress DENV2-mCherry infection with an mCherry-targeting crRNA (mCh3 crRNA; Table S1) and was chosen for subsequent experiments (Figure S1B). Though the site of flaviviral RNA replication is associated with ER membrane,^19^ localizing PspCas13b to ER with tail-anchor sequences such as SQS or VAMP2^20^ failed to suppress DENV2-mCherry infection (Figure S1B). The suppression of DENV2-mCherry by mCh3 crRNA was specific as other reporter DENV2 could not be suppressed by the crRNA in both single-virus infection and co-infection settings (Figures S1C and S1D). Using BHK-21-Cas13b, we individually tested 51 crRNAs against the targets on DENV2 reporter viruses, DENV2-16681 (NS5 gene), and ZIKV-SV0010 (NS2A gene; Table S1). While half of the crRNAs targeting fluorescent reporter genes (6 out of 12) were effective at viral suppression, only a small fraction of tiled crRNAs targeting the viral genes (3 out of 39; Figure S1E) could efficiently suppress virus (relative MFI < 0.4). We further validated the most efficient DENV2-targeting crRNA, 8681 crRNA, with DENV2-16681. We found that it could reduce virus titer between 10- and 15-fold relative to the nontarget crRNA at 24–48 hours post infection (hpi) and was specific to DENV2-16681 as nontarget ZIKV-SV0010 was not affected (Figure S1F). Infected BHK-21-Cas13b cells with 8681 crRNA were still actively dividing at 72 hpi (and potentially maintaining the infectious titer in the media) while most BHK-21-Cas13b with mCh3 crRNA were dead from infection with dropping titer (Figure S1F, bright-field image panels). Thus, PspCas13b with 8681 crRNA could retard DENV2 infection and protect host cells from its cytopathic effect in the absence of functional host antiviral response.

### Characterization of Cas13b RNP delivery by VLP

**Figure 1:**
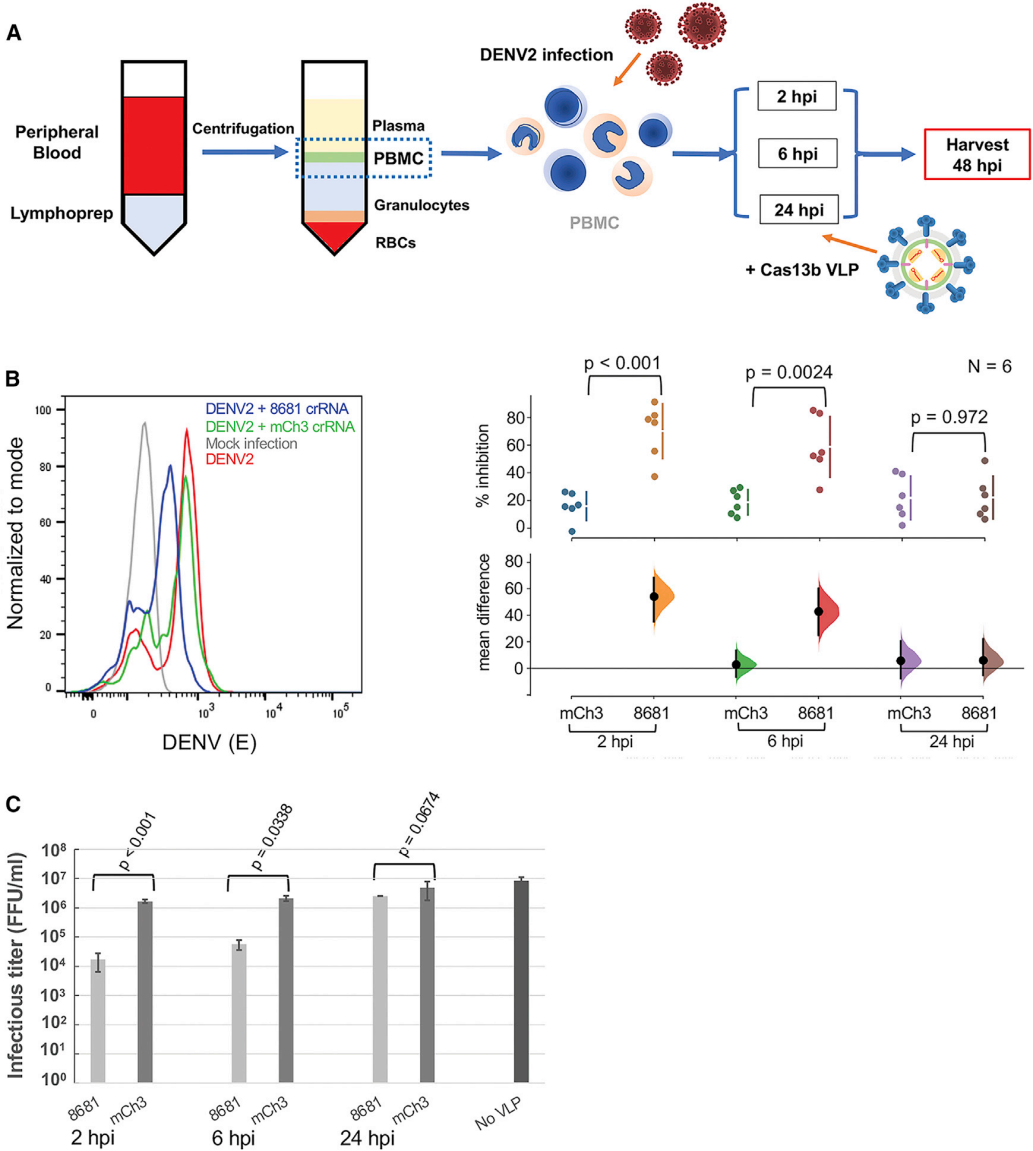
Efficient suppression of DENV2 infection by PspCas13b RNP delivered via virus-like particles (VLPs) (A) Diagram describing the experimental setup of Cas13b RNP delivery by VLP into human PBPMCs infected with DENV2-16681. (B) The reduction of infection percentage by PspCas13b + 8,681-nt crRNA against DENV2-16681 in PBMCs. Cas13b RNP was delivered by VLP at 2, 6, or 24 hours post infection (hpi). The left histogram is a representative result from PBMCs of one donor at 2 hpi VLP delivery. DV, the level of dengue E antigen measured by anti-E 4G2 mAb. The right Cumming plot displays DENV2 suppression with 8681 crRNA and mCh3 crRNA (nontarget control). The mean differences for 5 comparisons against the shared control mCh3 crRNA, 2 hpi are shown in the above Cumming estimation plot. The raw data are plotted on the upper axes (sample size = 6 for each condition). On the lower axes, mean differences are plotted as bootstrap sampling distributions. Each mean difference is depicted as a dot. Each 95% confidence interval is indicated by the ends of the vertical error bars. The VLPs were delivered with equal Cas13b dose of 1,720 ng/100,000 cells. The percent inhibition was calculated by the frequency of infected cells with the VLP divided by the frequency of infected cells without VLP. The results represent the data from three donors and duplicate experiments. (C) The reduction of infectious titers by PspCas13b + 8681-nt crRNA against DENV2-16681 in BHK-21 cells. The results are from triplicate experiments. The infections were performed as detailed in (A) with MOI = 1. Error bar, standard deviation.

Despite a relatively high and stable expression of CRISPR-Cas13b in our setup (an estimate of 994 ng/100,000 cells when induced with doxycycline at 0.1 μg/mL), delivery needs to be improved for practical use. Lentivirus delivery of CRISPR-Cas13 expression cassettes into cells has several limitations. It requires transcription and translation of CRISPR-Cas13 before the effector becomes available to target viral RNA, a process that requires several hours and could affect the effectiveness of virus suppression. CRISPR-Cas13 could also be permanently engrafted into the host genome, a process that carries the risks of transforming host cells and Cas13-toxicity from prolonged expression. Recently, retrovirus has been re-engineered to deliver protein cargoes and Cas9-gRNA RNP into cells in the form of VLP.^21^^,^^22^ Cas13 RNP delivery by VLP allows for immediate targeting of virus and avoids genotoxicity. To produce VLP for Cas13b RNP delivery, we fused PspCas13b gene to GAG gene to construct GAG-PspCas13b plasmid and co-transfected it with crRNA, Gag-Pol, and vesicular stomatitis virus G protein (VSV-G) plasmids into 293T (Figure S2A). We first tested VLP delivery of yellow fluorescent protein (YFP) into various dengue natural target cells that include primary human cells such as human dendritic cells (hDCs), macrophages, CD14^+^ monocytes,^23^^,^^24^ and hepatocytes^25^ (iMHC^26^). The VLP was effective at delivering YFP to these cells (Figure S2B). We found that the VSV-G pseudotyped VLP preferentially targeted CD14^+^ monocytes in human peripheral blood mononuclear cells (PBMCs) over B cells and T cells (Figure S2C). VLP delivery of PspCas13b with 8681 crRNA (Figure 1A) could suppress DENV2-16681 infection in human PBMCs (Figure 1B). The deliveries of 1,720 ng of Cas13b/100,000 cells (or 22 nM) at 2 and 6 hpi were able to reduce dengue infection by at least half over the infection course of 48 h (Figure 1B). However, the delivery at 24 hpi failed to reduce infection (Figure 1B). The same experiment in BHK-21 also showed similar effect of delivery time on the reduction of infectious titer (Figure 1C). Together, these results show that VLP can be an effective way to deliver antiviral Cas13b RNP to reduce dengue infection, but the efficacy depends on the delivery time.

### Shortening spacer length could improve knockdown activity of PspCas13b

**Figure 2:**
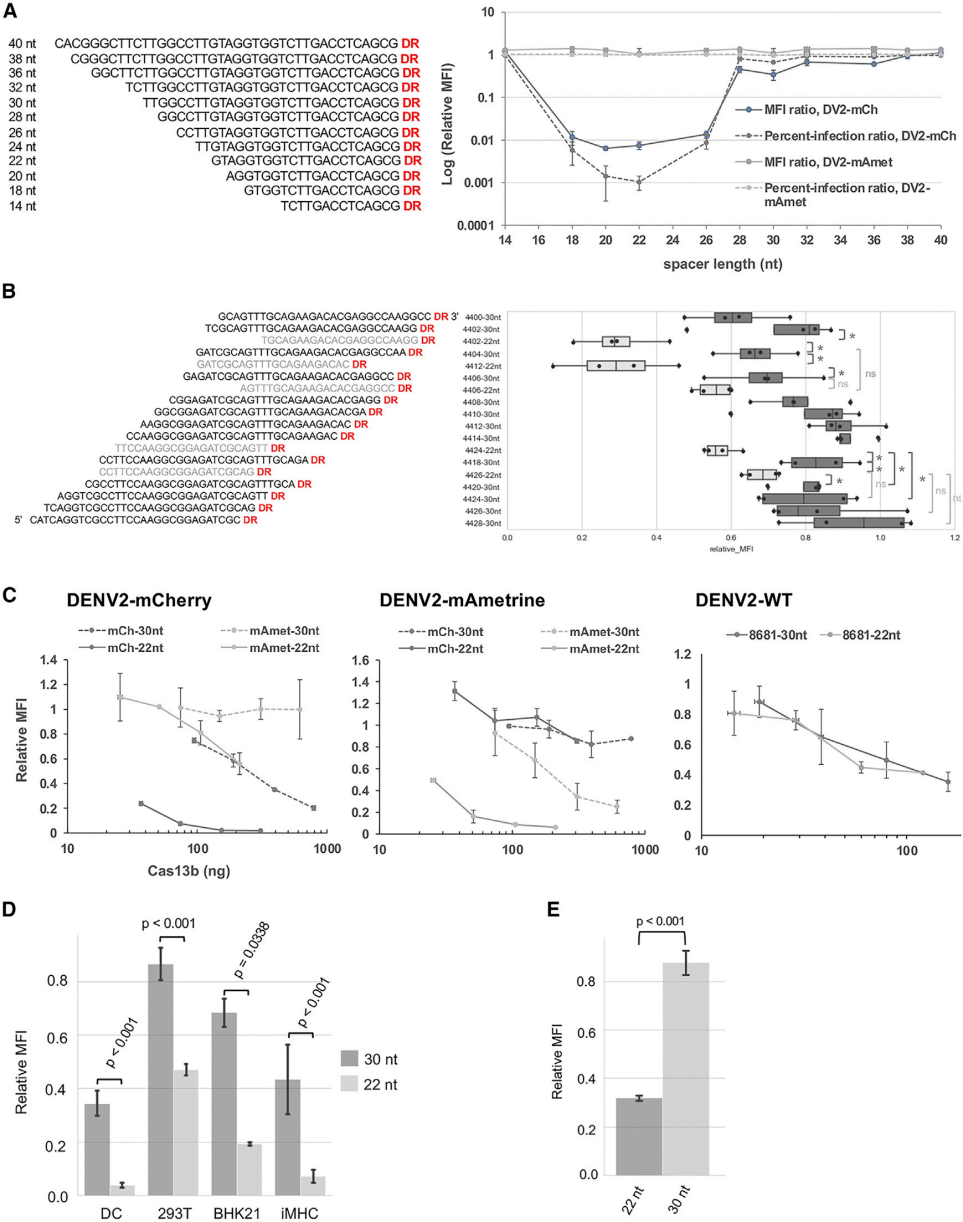
The effect of spacer length on the knockdown activity by PspCas13b (A) The effect of spacer length on the antiviral activity of mCh3 crRNA against DENV2-mCherry and DENV2-mAmetrine. The left panel shows the tiled spacer sequences of mCh3 crRNAs. The right plot displays the knockdown level of each DENV2 reporter with mCh3 crRNA of different spacer length. The measurements were performed in triplicate. (B) crRNAs with 22 nt spacer could enhance the accessibility of a target region on ZIKV NS2A gene. The data points were the averages of an experiment (performed in duplicate or triplicate). Statistical analyses were done with four averages per condition. ∗p < 0.05 and ns = p > 0.05. (C) Dose-response curve of Cas13b RNP delivered by VLP in BHK-21 cells for three pairs of 22-nt versus 30-nt crRNAs (mCh3, mAmet1, and 8681). The measurements were done in duplicate. (D) The enhancement effect by 22-nt spacer on the antiviral activity of the mCh3 crRNA was observed in multiple cells such as 293T, BHK-21, hDC, and iMHC. The measurements were done in triplicate. (E) The enhancement effect by 22-nt spacer of the mCh3 crRNA on knockdown of overexpressed mCherry reporter gene. The measurements were done in four replicates. Error bar, standard deviation.

Our results in Figure S1E also suggested that the knockdown efficiency could be improved for several targets. Using BHK-21-Cas13b, we tested whether spacer length could affect viral suppression efficiency for a subset of these crRNAs. Strikingly, shortening spacer length to 18–26 nts of mCh3 crRNA could drastically enhance the suppression of DENV2-mCherry with maximum knockdown activity achieved between 20 and 22 nts and without cross-suppressing nontarget DENV2-mAmetrine (Figure 2A). We also found that 22-nt spacer length could improve targeting efficiency against a ZIKV NS2A region compared to crRNAs with 30-nt spacer (crRNA) tiled against it (Figure 2B). Shortening spacer enhanced the knockdown activity of several crRNAs with varying efficiency (Figures S3A and S3B). Nevertheless, the knockdown activities of two crRNAs (mAmet1 and 8681) were aggravated by 22-nt spacer (Figures S3A and S3B). We selected three pairs of 30-nt versus 22-nt crRNAs for testing by VLP delivery in BHK-21 cells. The knockdown enhancement was recapitulated with Cas13b RNP delivery by VLP for 22-nt versus 30-nt mCh3 crRNAs (Figure 2C). In contrast to the results in BHK-21-Cas13b, the 22-nt mAmet-1 crRNA could knock down DENV2-mAmetrine with much less PspCas13b than its 30-nt counterpart (Figure 2C). The dose-response curves indicated that the 22-nt mCh3 and mAmet1 crRNAs needed 15- to 20-fold less of PspCas13b than 30-nt crRNAs to achieve the same level of virus suppression (Figure 2C). 22-nt 8681 crRNA also did not show any deterioration of knockdown activity as observed in BHK-21-Cas13b (Figure S3B; Figure 2C). These results together show that short spacer length could enhance the antiviral activity of PspCas13b and did not compromise its specificity.

We tested whether the enhancement effect of short spacer could be observed in different settings. VLP delivery of Cas13b with 22-nt mCh3 crRNA to 293T, hDC, and iMHC showed superior knockdown activity over its 30-nt counterpart with Cas13b dose at 2,575 ng/100,000 cells (Figure 2D), indicating that the effect was not cell-specific. In BHK-21-Cas13b, 22-nt mCh3 crRNA could knock down overexpressed mCherry reporter gene while 30-nt crRNA could not (Figure 2E), suggesting that the effect was applicable to regular mRNA. Interestingly, *in vitro* cleavage of mCherry RNAs by PspCas13b was aggravated by 22-nt crRNA (Figure S3C), suggesting that the effect was specific to mammalian cells.

### Short spacer length did not affect crRNA processing by PspCas13b

**Figure 3:**
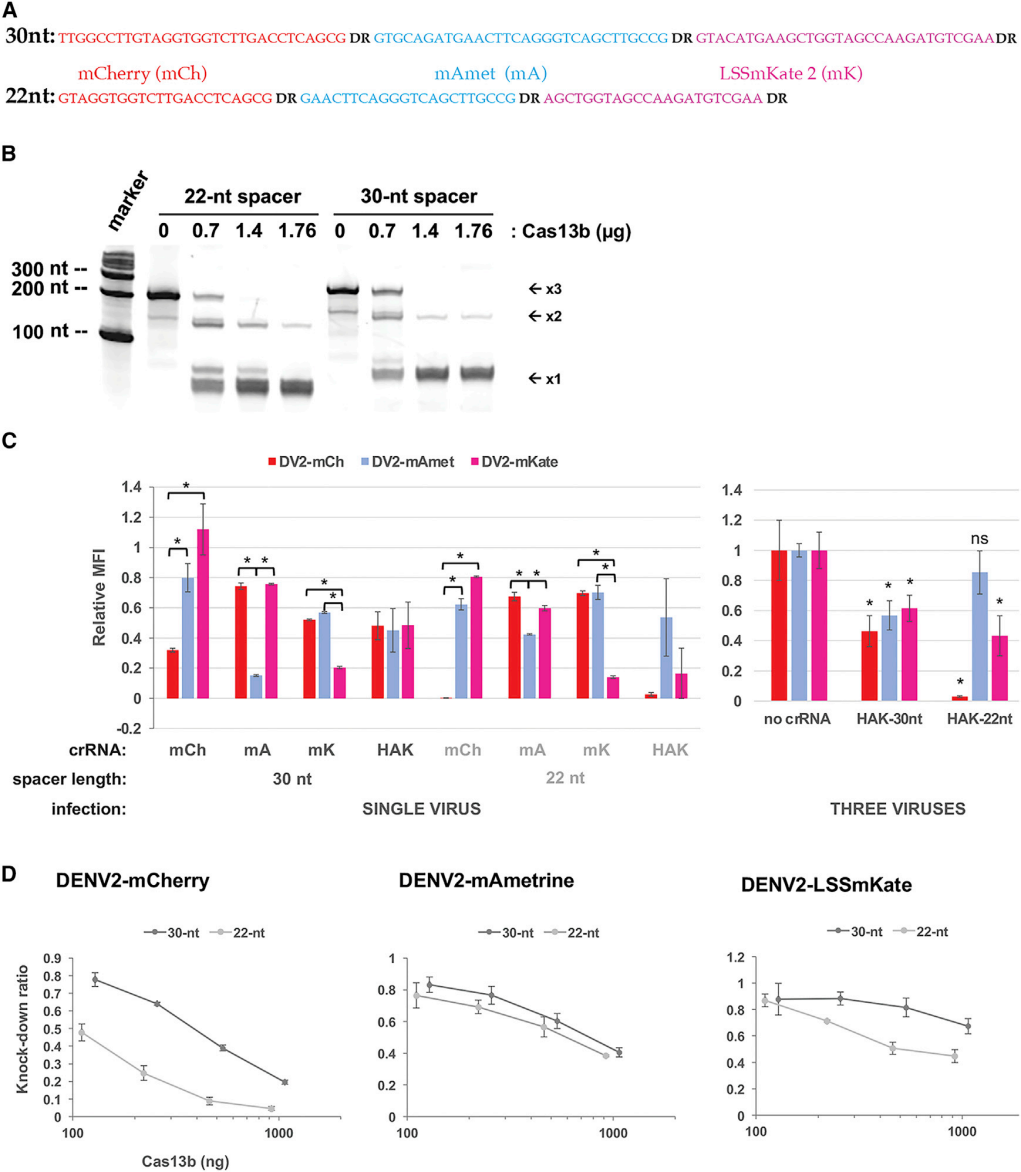
Short spacer did not inhibit crRNA processing and multiplex targeting of PspCas13b by a crRNA array (A) The sequences 30-nt and 22-nt crRNA arrays used for testing *in vitro* crRNA processing and multiplex targeting. DR, direct repeat. (B) *In vitro* crRNA processing analyzed by 8 M urea PAGE. 3x, crRNA array with three crRNAs, 2x, crRNA array with two crRNAs, 1x, singlet crRNAs. (C) Targeting of multiple reporter DENV2 with the crRNA array in single-virus (left bar plot) and three-virus (right bar plot) infections. HAK, crRNA array shown in (A). The measurements for crRNA arrays were done in four replicates. For three-virus infections (right bar plot), p values were calculated from side-by-side comparison between no crRNA and HAK with the same reporter DENV2. ∗p < 0.05, ns, p > 0.05. (D) Dose-response curves of multiplex VLP-Cas13b RNP generated with a crRNA array. The measurements were done in duplicate. Error bar, standard deviation.

Since natural spacer lengths found in crRNA array of CRISPR-Cas13b in bacteria are no shorter than 30 nts,^6^^,^^27^ we tested whether 22-nt spacer could affect crRNA processing and multiplex targeting by a crRNA array (Figure 3A). *In vitro* crRNA processing of 22-nt and 30-nt arrays with PspCas13b was equally efficient (Figure 3B). Multiplex targeting of fluorescent reporter DENV2 could also be achieved with 22-nt crRNA array in both BHK-21-Cas13b (Figure 3C) and in VLP formats (Figure 3D). Multiplex targeting by both 30-nt and 22-nt crRNA arrays reduced knockdown efficiency for each reporter virus slightly compared to the single-virus targeting by single crRNAs (Figure 3C, left bar plot). 22-nt crRNA array generally suppressed reporter DENV2 more than 30-nt crRNA array in both single-virus (Figure 3C, left bar plot) and triple-virus (Figure 3C, right bar plot) infections. Dose-response curves of multiplex VLP generated with a crRNA array showed that more Cas13b was required to achieve the same level of suppression compared to Cas13b-single crRNA by 3- to 10-fold (Figure 3D versus 2C). The knockdown activities by multiplex VLP against reporter DENV2 generally conformed to what had been observed with singlet VLPs, though the enhancement effect of 22-nt spacer for mAmeterine was drastically reduced in the multiplex targeting (Figure 3D versus 2C).

## Discussion

In summary, we characterized the antiviral efficiency of CRISPR-Cas13b in suppressing DENV2 and ZIKV infection in mammalian cells (Figure S1). We showed that transient Cas13b RNP delivery by VLP could effectively suppress dengue infection in both cell lines and primary human target cells (Figures 1 and 2D). We found that shorter spacer length could significantly improve the knockdown activity of CRISPR-Cas13b in mammalian cells and increased the number of targetable sites (Figure 2). The short spacer length (22-nt) did not affect crRNA processing and still enabled multiplex targeting with crRNA array (Figure 3).

Our results demonstrated the effectiveness of transient delivery of PspCas13b RNP by VLP in reducing dengue infection in primary human target cells. VLP delivery of Cas13b RNP delivery could negate several limitations such as genotoxicity and gene-size restriction imposed by gene delivery vectors such as lentivirus and AAV. Previous studies have shown that Cas13 RNP or RNA delivery could suppress RNA virus infection but the delivery methods by electroporation and transfection are not suitable for *in vivo* and could be highly toxic to primary cells.^1^^,^^10^ Our results show that VLP delivery could effectively deliver Cas13b RNP into a variety of cells without high toxicity. Our results showed that with the right crRNAs, a picomolar range of PspCas13b could achieve strong suppression of dengue infection (e.g., 30 ng of PspCas13b per 100,000 cells [or 452 pM of PspCas13b] with 22-nt mCh3 crRNA and 22-nt mAmet1; Figure 2C). Nevertheless, there are two potential limitations that still need to be addressed. First, in the case of DENV, the antiviral efficacy of Cas13b-VLP could be compromised when administered to infected cells between 6 and 24 hpi (Figures 1B and 1C). Second, as VLP-delivered Cas13b and crRNA will not amplify in the infected cells, repeated treatment may be needed. Host immune responses to the first-time VLP delivery may compromise further treatment using the same VLP system. Additional engineering on both Cas13b and VLP could provide solutions to these issues. Protein engineering on Cas13b may improve its antiviral activity. The surface of retrovirus/lentivirus VLP could accommodate more host proteins to modulate the interactions with the host immune system, providing flexibility for engineering.^28^ Gene editing of the producer cells can also be used to eliminate specific surface proteins on the virus particle.^29^

Our results highlight a distinct difference between CRISPR-Cas13b RNA targeting in mammalian cells and *in vitro*/bacteria. Consistent with our study, shortening spacer length between 18 and 26 nts did not appear to reduce PspCas13b binding to target RNA in mammalian cells as shown by previous live-imaging study using dCas13b-fluorescent fusion proteins.^30^ In contrast, our results of *in vitro* cleavage by PspCas13b (Figure S3C) and previous characterizations of other Cas13b by *in vitro* cleavage and in bacteria showed that optimal spacer length coincided with natural spacer length of 30 nts.^27^^,^^31^ In contrast to Cas13b and Cas13a, Cas13d naturally utilizes varying spacer lengths and is not affected by short spacer length in all settings.^8^^,^^14^^,^^32^ Despite the general benefits of short spacer lengths for PspCas13b in mammalian cells, there were cases where they aggravated knockdown activities. These exceptions were noticed only in the setting of overexpressed Cas13b and crRNA in BHK-21-Cas13b (Figure S3), but not in the setting of VLP delivery (Figure 2C). There were notable differences between the two delivery methods such as the dosage and the dynamics of Cas13b and crRNA in the cells. In VLP delivery, crRNA was pre-assembled with Cas13b before introduction into target cells while unbound crRNA could be present in the overexpression setting. In VLP delivery, the CRISPR-Cas13 components may stay only in the cytoplasm of targeted cells while crRNA in overexpression setting were produced in nucleus of targeted cells. Additional investigations will be needed to understand how these differences could affect the RNA targeting by CRISPR-Cas13b as they are relevant for designing effective delivery strategies.

Previous crRNA-scanning studies have shown that only a small fraction of target regions on mRNA in mammalian cells was targetable by CRISPR-Cas13.^1^^,^^7^^,^^14^ These studies utilized a tiling crRNA library with a fixed spacer length to reveal the factors that determined the knockdown efficiency of a crRNA. Our results (Figure 2; Figure S3) suggest that varying spacer length in a scanning analysis could improve the number of targetable regions for CRISPR-Cas13b and might uncover additional factors that determine the knockdown efficiency of a crRNA. Combining this strategy with targeting conserved sites on viral RNA^1^^,^^9^ has the potential to identify highly potent antiviral crRNAs.

In addition to CRISPR-Cas13, RNAi has provided a programmable antiviral strategy for RNA viruses, with several antiviral RNAi therapeutics already in phase II trials.^33^ Fundamental differences existing between the two platforms need to be considered for antiviral development. For CRISPR-Cas13, both Cas13 protein and crRNA needs to be delivered into mammalian cells to realize viral RNA targeting. For RNAi, only small interfering RNA (siRNA) or short hairpin RNA (shRNA) are delivered to guide endogenous RNAi machinery to target viral RNA. An advantage of exogenous CRISPR-Cas13 is the possibility of engineering both Cas13 proteins and crRNA to improve antiviral activity. As host RNAi machinery has complex functions in controlling host gene expression, engineering the RNAi protein components to improve antiviral activity is prohibitively complicated. Although immune response against exogenous Cas13 protein could reduce its therapeutic activity in repeated dosing, the diversity of CRISPR-Cas13 and recent advances in protein engineering may help alleviate this issue.^34^, ^35^, ^36^ Viral proteins of several human RNA viruses and interferon stimulated genes (ISGs) were shown to dampen RNAi activity.^37^ In contrast, since CRISPR-Cas13 is of bacterial origin, it is likely that mammalian viruses have never interacted with it in nature and are likely not able to counter it right away.

Overall, our study addressed two key aspects of the implementation of antiviral CRISPR-Cas13 against RNA viruses. We showed that VLP was an effective vehicle to deliver PspCas13b RNP into a variety of primary cells. We showed that crRNA with short spacer length (~20–26 nt) could improve knockdown activity of CRISPR-Cas13 in mammalian cells.

## Materials and methods

### Cells and culture media

BHK-21, BHK-21-rtTA3,^17^ and 293T were cultured in high-glucose DMEM (HyClone) supplemented with 10% heat-inactivated fetal bovine serum (HI-FBS, ThermoFisher Scientific), 100 U/mL penicillin-G, and 100 μg/mL streptomycin sulfate (ThermoFisher Scientific; D10). Vero cells was maintained in MEM (ThermoFisher Scientific) supplemented with 10% HI-FBS and 100 U/mL penicillin-G, and 100 μg/mL streptomycin sulfate (MEM10). iMHC was maintained in DMEM-F12 (Hyclone) supplemented with 10% HI-FBS. C6/36 was maintained in Leibovitz L-15 (HyClone) supplemented with 10% HI-FBS, 10% tryptose phosphate broth (Sigma), and 100 U/mL penicillin-G, and 100 μg/mL streptomycin sulfate. Human primary PBMCs, macrophages, and CD14^+^ monocytes were maintained in RPMI-1640 (HyClone) supplemented with 10% HI-FBS, 100 U/mL penicillin-G, and 100 μg/mL streptomycin sulfate (R10). All the adherent cells in this study were detached by 0.1% Trypsin-EDTA (ThermoFisher Scientific). All mammalian cells were cultured under 5% CO_2_ at 37°C with at least 80% humidity. C6/36 was cultured at 28°C.

Primary human PBMCs were obtained from fresh blood of healthy volunteers. Human blood was obtained from donors after providing informed consent, following a protocol (Siriraj-IRB COA number Si707/2016, Protocol number: 632/2559 [EC2]) approved by Faculty of Medicine, Siriraj Hospital, Mahidol University, Thailand. The blood was diluted to a 1:1 ratio of normal saline and layered over Lymphoprep (Axis-shield) gradient density at 1,077 g/mL. After centrifugation at 800 × *g* for 25 min (min) at room temperature (RT), plasma samples were removed from the top of the solution and PBMCs were recovered from the underlying layer. The PBMCs were washed twice in RPMI 1640 medium and then incubated with red blood cells lysis buffer at RT for 5 min to lyse contaminating RBCs. The PBMCs were washed once in R10. PBMC viability, measured by trypan blue exclusion, was greater than 95%.

hDCs were derived from PBMC according to published protocol.^38^ 1.5 × 10^7^ of PBMCs were seeded on each 60 mm-Primaria plate (Corning) and cultured for 1 h. Cells were washed with 5 mL of plain RPMI 10 times to remove non-adherent cells.^38^ Washed PBMCs were then replenished with 5 mL of R10 supplementing with 2,000 U/mL of recombinant human (rH) GM-CSF (ThermoFisher Scientific) and 4,000 U/mL of rH interleukin-4 (IL-4; R&D Systems) and cultured for 5 days. DCs were then harvested from culture supernatant for experiments and analysis. DCs were verified with a panel of CD14-PerCP (Miltenyl Biotech), CD83-BV510 (BD PharMingen), CD86-PE (BD PharMingen), HLA-DR-APC-Vio770 (Miltenyl Biotech), CD209-PE-Vio770 (Miltenyl Biotech), and CD163-FITC (BioLegend) monoclonal antibodies (mAbs) by flow cytometry (BD LSRFortessa).

Fresh PBMCs were used to isolate primary monocytes using Dynabeads Untouched Human Monocytes (ThermoFisher Scientific) according to the manufacturer’s instructions. Briefly, the PBMCs were incubated with blocking reagent and antibody mixture for 20 min at 2°C to 8°C to label cells that were not monocytes. The labeled cells were mixed with Dynabeads and incubated for 15 min at 2°C to 8°C. To remove non-monocytes, we placed the labeled cells in a magnet (DynaMag). The purity of the isolated monocyte subsets, evaluated by flow cytometry with CD14-PerCP mAb (Miltenyl Biotech), was consistently 90% to 95% of CD14^+^ cells.^38^

The isolated CD14^+^ monocytes were cultured in R10 supplemented with 5% HI-human AB serum to allow differentiation into macrophages.^39^ Half volume of medium was replaced with fresh medium every 3 days. The macrophages were harvested for experiments on day 13. The macrophages were then verified by flow cytometry analysis with CD163-FITC mAb (BioLegend).

### Plasmids

Spacer sequences used to construct crRNAs are listed in Table S1 and Figures 2 and S3. All the plasmids were constructed either by Gibson assembly or by standard T4 ligation.

PspCas13b from pC0046^6^ (Addgene #103862) was subcloned to replace CasRx on pXR001^8^ (Addgene #109049). The pXR001-PspCas13b was then engineered to replace HIV Rev nuclear export signal (NES) with VAMP2 (amino-acid sequence = KTGKNLKMMIILGVICAIILIIIIVYFTGSR) or SQS (amino-acid sequence = SRSHYSPIYLSFVMLLAALSWQYLTTLSQVTED) to generate lentiviral plasmids for PspCas19-VAMP2 and PspCas13-SQS. These pXR001-Cas13b plasmids were used for the experiment in Figure 1B.

To create lentiviral vector with inducible expression of Cas13b, we subcloned PspCas13b from pC0046^6^ (Addgene #103862) into pENTR1A^40^ (Addgene #17398) at EcoRI site. PspCas13b on pENTR1A was shuttled to pLenti-CMVtight-Blast-DEST^40^ (Addgene #26434) with Gateway cloning (ThermoFisher Scientific). Blasticidin-resistant gene was then replaced with eGFP for the purpose of cell sorting. This pLenti-PspCas13b plasmid was used to generate lentivirus for deriving BHK-21-Cas13b stable cell line.

pBA439^41^ (Addgene #85967) was engineered to replace Cas9-gRNA cassette with Psp direct repeat (DR) with BsmBI sites upstream for cloning spacer and to replace BFP with miRFP703 for the purpose of cell sorting. The lentiviral plasmid, pBA439-Psp-miRFP, was then used for testing crRNAs in BHK-21-Cas13b.

To create plasmid for PspCas13b RNP delivery by VLP, we engineered BIC-Gag-CAS9^22^ (Addgene #119942) to replace Cas9 with PspCas13b-HA to generate BIC-Gag-PspCas13b. pC0043 (Addgene #103854) was used to clone spacer to generate a crRNA for VLP production.

PspCas13b on pC0068 plasmid (Addgene # 115219) was replaced with PspCas13b with HIV NES and hemagglutinin (HA) tag (from pC0046; Addgene #103862) to generate pC0068-PspCas13b-HA for PspCas13b-HA purification.

crRNA arrays (mCh3-mAmet1-mK1) were generated by T4 ligation of 3 pairs of annealed primers with asymmetric, unique overhangs to pBA439-Psp-miRFP linearized with BsmBI (to generate pBA439-HAK) for experiments in BHK-21-Cas13b. The crRNA arrays were then subcloned onto pC0043 linearized with BbsI and XhoI to generate pC0043-crRNA array plasmid for multiplex VLP generation.

### Viruses

DENV2-16681 and ZIKV-SV0010/15 were produced from C6/36 cell line cultured in L-15 (HyClone) supplemented with 1.5% HI-FBS (ThermoFisher Scientific), 10% tryptose phosphate, and 100 U/mL penicillin-G, and 100 μg/mL streptomycin sulfate. The infectious titers were quantitated by foci assay in Vero cells stained with anti-E 4G2 monoclonal antibody as previously described.^17^ Fluorescent reporter DENV2s were generated and quantitated as previously described.^17^

### Lentivirus for gene delivery

Pseudotyped lentivirus particles were generated by co-transfection of lentiviral plasmids (e.g., pBA439-Psp-miRFP and pLenti-CMVtight-PspCas13b-eGFP) with pCMV-VSV-G (Addgene #8454) and psPAX2 (Addgene #12260) using PEI into 293T cells cultured in D10.^42^ The media were harvested for lentivirus particles 2 days post transfection.

### Immunofluorescent microscopy

Briefly, cells were seeded in a 96-well plate to reach 50%–60% confluency overnight. Cells were washed and fixed with 100 μL 4% paraformaldehyde in PBS for 10 min at 37°C, permeabilized with 100 μL 1% Triton X-100 in 1× PBS for 15 min at 37°C, and blocked with 1% BSA in 1× PBS for 1 h at 37°C. Cells were then incubated with primary antibody (HA-Tag Mouse mAb, 1:100 dilution; Cell Signaling), washed and incubated with secondary antibody (Cy3 conjugated goat anti-mouse, 1:2,000 dilution; Jackson ImmunoResearch). Samples were washed and images were obtained from an EVOS fluorescence microscope (ThermoFisher Scientific).

### Testing crRNAs with Cas13b inducible expression system in BHK-21

BHK-21-rtTA3 cell line^17^ was transduced with lentivirus generated from pLenti-PspCas13b. Transduced cells (GFP^+^) were sorted into single cells to isolate stable clones on BD FACSAria III. To screen for desired clone (BHK-21-Cas13b), we tested the clones for uniform Cas13b expression and their abilities to knock down DENV2-mCherry with mCh3 30-nt crRNA. A BHK-21-Cas13b clone was selected for all subsequent experiments. A crRNA was introduced into the BHK-21-Cas13b clone with the lentivirus carrying mU6-crRNA expression cassette and miRFP703-2A-puromycin cassette for cell sorting. Cells that were positive for both GFP (PspCas13b) and miRFP703 (crRNA) were sorted as a pool. The pool cells were maintained for at least a week under D10 + 5 μg/mL puromycin before the knockdown experiments. To test for knockdown, we seeded 100,000 cells/well in 6-well plate with D10 + 0.1 μg/mL of doxycycline to induce Cas13b expression. After 24 h, the induced cells were then infected with DENV2 or ZIKV at MOI = 0.1 to test for knockdown activity of the crRNA. The cells were maintained at 37°C for 72 days before harvested by trypsin digestion and fixed with 3.7% formaldehyde in 1× PBS for measurement of virus infection by flow cytometry (BD LSRFortessa).

Virus infection was measured by mean fluorescent intensity (MFI) of fluorescent reporters (for reporter DENV2 such as DENV2-mCherry, DENV2-mAmetrine, and DENV2-LSSmKate2) or viral E antigens (stained with 4G2 mAb for DENV2-16681 and ZIKV-SV0010/15) of total cells or percent infection of total cells. For the knockdown ratio (KD ratio), MFI (or percentage of infection of total cells) of BHK-21-Cas13b with an experimental crRNA was divided by the MFI (or percent infection of total cells) of BHK-21-Cas13b with a non-targeting crRNA.

### Production of VLP-YFP and VLP-Cas13b RNP

17,000,000 293T cells were seeded in a 150-mm dish. To generate VLP-Cas13b RNP, we transfected one dish of 293T with four plasmids as follows: 5.1 μg BIC-Gag-PspCas13b, 13.2 μg pC0043-crRNA, 3.3 μg pCMV-VSV-G^42^ (Addgene #8454), and 8.4 μg pBS-CMV-gagpol (Addgene #35614) using 90 μg 1 mg/mL PEI (3 μg PEI: 1 μg total DNA) 24 h after seeding. For VLP-YFP, BIC-Gag-PspCas13b and pC0043-crRNA were replaced with 5.1 μg MLV-Gag-YFP^43^ (Addgene #1813). The transfected cells were maintained for 2 days before harvest of media. Harvested media was clarified by centrifugation at 1,000 × *g* at 4°C for 10 min and then filtered with 0.45-μm PES-membrane syringe filter (Millipore). The VLP in the media was concentrated by one of the two methods. In the first method, 30 mL of clarified media was then layered on 10 mL of 10% w/v sucrose cushion in 1× PBS in 50 mL falcon tube (Corning) and centrifuged at 10,000 × *g* at 4°C for 4 h in JA14 rotor. The supernatant was carefully discarded and dried by pressing against paper towel for 30 s. The pellet was resuspended with D10. Dissolved pellet was aliquoted and stored frozen at −70°C. In the second method, 7.5 mL of PEG solution (PEG Virus Precipitation Kit, Abcam) was mixed with 30 mL of clarified media and incubated at 4°C overnight. Centrifugation was performed at 3,200 × *g* at 4 °C for 30 min. Then supernatant was discarded and VLP pellet was re-suspended with 500 μL re-suspension solution (PEG Virus Precipitation Kit, Abcam). Dissolved pellet was aliquoted and stored frozen at −70°C.

### Purification of PspCas13b

PspCas13b-HA was expressed and purified as previously reported with some modifications.^31^ PspCas13b-HA was expressed in BL21-Rossetta2 by 2 mM IPTG induction in 2xYT media at O.D. of 0.6, 22°C for 18 h. The bacteria cells were harvested by centrifugation and stored at −70°C until purification. The bacteria pellet was resuspended in 20 mM Tris-HCl pH 8.0, 500 mM NaCl, 1 mM DTT + protease inhibitors (EDTA-free, Roche) + lysozyme, lysed by sonication, and clarified by centrifugation at 10,000 x g, 4°C for 20 min. Clear lysate was loaded onto streptactin column. The column was then washed with 20 mM Tris-HCl pH 8.0, 500 mM NaCl, 1 mM DTT before elution with the same buffer + 0.15% NP-40 + 2.5 mM desthiobiotin. The eluted fraction was treated with Ulp1 protease to cleave affinity tag overnight at 4°C. Ulp1 and cleaved affinity tag were removed by NiNTA (Roche). The NiNTA flow through was adjusted to 250 mM NaCl and purified on HiTRAP-SP (20 mM HEPES pH 7.3, 250 mM NaCl, 5% glycerol, 1 mM DTT). We obtain highly purified PspCas13b-HA that was used as Cas13b reference for VLP quantitation and *in vitro* crRNA processing.

### Quantitation of Cas13b-VLP

We used two methods, dot-blot assay and ELISA, to quantitate Cas13b VLP. For dot-blot assay, 2 μL from serial dilutions of pure PspCas13b-HA and VLP were dotted on a strip of nitrocellulose membrane. The membrane was blocked with 5% skim milk in 1× PBS-T. The blocked membrane was then probed with anti HA-Tag (6E2) Mouse mAb diluted 1:1,000 (Cell Signaling) and rabbit anti-mouse P260 immunoglobulin G (IgG) conjugated with horseradish peroxidase (HRP; 1:1,000 dilution, Dako). The antibody-stained membrane was then soaked with SuperSignal West Pico PLUS substrate (ThermoFisher Scientific) according to the manufacturer’s instruction and imaged on C-Digit blot scanner (LI-COR). Quantitation of signal was performed with Image Studio Lite program (LI-COR). For ELISA, ELISA was carried out as previously described with slight modifications.^44^ Briefly, VLP and Cas13 protein standard were treated with SDS at the final concentration of 2% for 1 h at 37°C. The proteins and the VLP were serially diluted in carbonate buffer (0.05 M, pH 9.6) before being used to coat in ELISA microwell plates (Maxi Sorp, Nunc) and incubated for 1 h at 37°C. After saturating microwell plates with 1% BSA (Sigma-Aldrich) for 1 h, the wells were washed five times with 1× PBS containing 0.05% Tween 20. Then anti HA-Tag (6E2) Mouse mAb (Cell Signaling) diluted 1:1,000 was added, and the plates were incubated for 1 h at 37°C. Washing was performed as above. Rabbit anti-mouse IgG conjugated with HRP (P260, Dako) diluted 1:1,000 was added, and the plates were incubated for 1 h at 37°C, followed by five washes. After 50 μL of TMB substrate solution was added (ThermoFisher Scientific), the reaction was quenched by adding 50 μL of stop solution. The absorbance of the plates was measured at 450 nm using a microplate reader (Tecan Sunrise).

### Delivery of protein cargo by VLP

VLP delivery media was prepared by mixing a volume of VLP preparation according to the desired Cas13b dose and topped up with D10 to 100 μL. For VLP-YFP, 50 μL of VLP preparation was mixed with 50 μL D10. To deliver VLP into BHK-21, iMHC, and 293T cells, we treated the cells (BHK-21 ~100,000 cells; 293T and iMHC ~150,000–200,000 cells) in one well of 24-well plate with 300 μL of complete media + 8 μg/mL polybrene for 10 min at 37°C before adding 100 μL of VLP delivery media. For these cells, the VLP-treated cells were then maintained for 24 h at 37°C before exchanging media to 500 μL D10 for another 24 h before harvest for analysis. To deliver VLP into hDC, we centrifuged 50,000 cells of hDC at 450 × *g* for 5 min at 4°C and resuspended the cell pellet with 300 μK R10 + 8 μg/mL polybrene. 100 μL of VLP delivery media (R10) was added to hDC. The cells were then transferred to one well of 24-well plate and cultured for 48 h at 37°C before harvest. VLP delivery of YFP was performed with the same protocol as VLP delivery of Cas13b.

### Test of knockdown in hDC, 293T, and iMHC by VLP

For iMHC and BHK-21, 25,000 cells/well were seeded on 24-well plate. For 293T, 50,000 cells/well were seeded on 24-well plate. After 24 h, the cells were infected with DENV2-mCherry at MOI 0.1 for another 24 h. VLP was then delivered as detailed above. For hDC, cell suspension (~50,000 cells/20 μL) was mixed 30 μL of DENV2-mCherry (MOI = 1.0) and topped up with 50 μL R10 in 1.5 mL Eppendorf tube and incubated at 37°C for 2 h. Then, the cells were washed once with 50 μL R10 by centrifugation at 450 × *g* for 5 min at 4°C and proceeded to VLP delivery.

### Test of knockdown in BHK-21 with overexpressed mCherry reporter

BHK-21-Cas13b cell line was transduced with lentivirus carrying TRE-mCherry reporter cassette (generated from pLV-tetO-mCherry, Addgene #70273). The transduced cells were sorted for mCherry^+^ cells. The BHK-21-Cas13b-mCherry was then transduced with crRNA lentivirus and sorted for GFP^+^mCherry^+^miRFP703^+^ cells. The sorted cells were maintained in D10 + 5 μg/mL puromycin for 1 week before knockdown experiment. The knockdown was initiated by adding 0.1 μg/mL doxycycline and continued the culture for 48 h. The cells were harvested by trypsin digestion and fixed with 3.7% formaldehyde in 1× PBS for analysis by flow cytometry (BD LSRFortessa).

### DENV2 knockdown in infected human peripheral blood monocytes

PBMCs were obtained as detailed above. The experiments were performed in a 24-well plate with PBMCs seeded at 100,000 cells/well in R10. To infect PBMC with DENV2-16681, we incubated 0.1 mg/mL of purified 4G2 (*Flavivirus* E protein specific mAbs) with virus for 1 h at 37°C before adding to PBMCs at MOI of 1 in R10. PBMCs were infected for 2 h at 37°C. To deliver VLP, we removed 200 μL of culture media and added 100 μL of R10 with 8 μg/mL polybrene to the well. PBMCs were treated with polybrene at 37°C for 10 min before 100 μL of VLP delivery media was added. Infected PBMC were cultured for 48 hpi before harvest for analysis.

Harvested PBMCs were washed and stained with live dead dye (Invitrogen) according to the manufacturer’s instruction. Stained cells were fixed and permeabilized with 3.7% formaldehyde and 0.5% saponin, respectively. Cells were then intracellularly stained with 4G2 mAbs followed by rabbit anti-mouse IgG FITC (Dako). The cells were fixed with 1% formaldehyde and analyzed on flow cytometry (BD LSRFortessa). Data analysis was performed using FlowJo software version 10.1.

For testing the uptake of VLPs in PBMCs, cells were incubated with YFP-VLPs 50 μL for 48 h at 37°C. Cells were washed and surface stained with CD3 APC (BD PharMingen), CD14 PerCP mAb (BD PharMingen), and CD19 PE mAb (Dako) for 30 min. Thereafter, cells were washed, fixed with 1% formaldehyde, and analyzed on flow cytometry (BD LSRFortessa). Data analysis was performed using FlowJo software version 10.1.

### *In vitro* transcription and crRNA processing assay

DNA template for *in vitro* transcription of a crRNA array was amplified from pBA439-HAK by PCR using a forward primer with T7 promoter sequence and a reverse primer that included poly-T to terminate transcription. 0.5 μg of DNA template were transcribed in 100 μL reaction using 30 μg T7 RNAP, 2 mM NTPs, 40 U RNaseIN (Promega) in 50 mM Tris-HCl pH 7.5, 15 mM MgCl_2_, 5 mM DTT, and 2 mM spermidine at 37°C for 2 h. The transcription reaction was treated with 2 U of DNase I (ThermoFisher Scientific) at 37°C for 30 min and clean-up using QIAGEN RNA easy kit according to the manufacturer’s protocol. *In vitro* crRNA array processing was carried out in 10 mM Tris-HCl pH 7.5, 50 mM NaCl, 0.5 mM MgCl_2_, 20 U RNaseIN (Promega), 0.1% BSA for 30 min at 37°C, stopped by adding 1% SDS, 2× TBE-Urea gel loading buffer and denatured for 10 min at 95°C. Samples were then put on ice for 10 min before running them on an 12% TBE^–^ 8 M Urea polyacrylamide gel in 1× TBE buffer at 200 V for 40 min. Gel staining was carried out in 1× SYBR Gold in 1× TBE for 5 min and imaged on a gel doc system (Syngene).

### *In vitro* cleavage of target RNA by PspCas13b

Cas13b-cRNA RNP was formed by mixing 1 or 2 μM of crRNA with 1.8 μM of Cas13b in total volume 5 μL and incubated on ice 10 min. The preformed RNP was then added to 10 μL *in vitro* cleavage reaction that contained 20 ng of mCherry RNA, 1 μL of 10× Cut smart buffer (NEB), and 4 U of RNase IN and incubated at 37°C for 2 h. The reaction was stopped with 1% SDS and then extracted by phenol/chloroform for ethanol precipitation. The RNA pellet was resuspended with 1× RNA loading buffer (4 mM EDTA, pH 8.0, 2.7% formaldehyde, 20% glycerol, 7.7 M formamide, 80 mM MOPS, 20 mM sodium acetate, 0.025% [v/v] bromophenol blue) and heated at 95°C for 10 min and then put on ice for 2 min before loading on 8% TBE-polyacrylamide gel with 8 M urea.

### Statistical analysis

Estimation statistics were used to analyze the mean differences between different conditions.^45^ The analysis and drawing of Cumming plot (Figure 1B) were performed on https://www.estimationstats.com/. Significance tests for multiple two groups and shared-control groups were performed by one-way ANOVA. Nonparametric, two-sided permutation t test was used to calculate p values.

## Supplemental information

Document S1. Figures S1–S3 and Table S1

Document S2. Article plus supplemental information
