# Supplementary material for: Potent programmable antiviral against dengue virus in primary human cells by Cas13b RNP with short spacer and delivery by VLP
Source: Mol Ther Methods Clin Dev. 2021 May 1;21:729–40. doi: 10.1016/j.omtm.2021.04.014 (PMC8087611; doi:10.1016/j.omtm.2021.04.014)
Supplement: Document S1. Figures S1–S3 and Table S1 [file mmc1.pdf]

OMTM, Volume *21*

Supplemental information

Potent programmable antiviral against dengue virus in primary human cells by Cas13b RNP with short spacer and delivery by VLP

Ekapot Singsuksawat, Suppachoke Onnome, Pratsaneeyaporn Posiri, Amporn Suphatrakul, Nittaya Srisuk, Rapirat Nantachokchawapan, Hansa Praneechit, Chutimon Sae-kow, Pala Chidpratum, Khanit Sa-ngiamsuntorn, Suradej Hongeng, Panisadee Avirutnan, Thaneeya Duangchinda, and Bunpote Siridechadilok

Supplementary Figures and Table

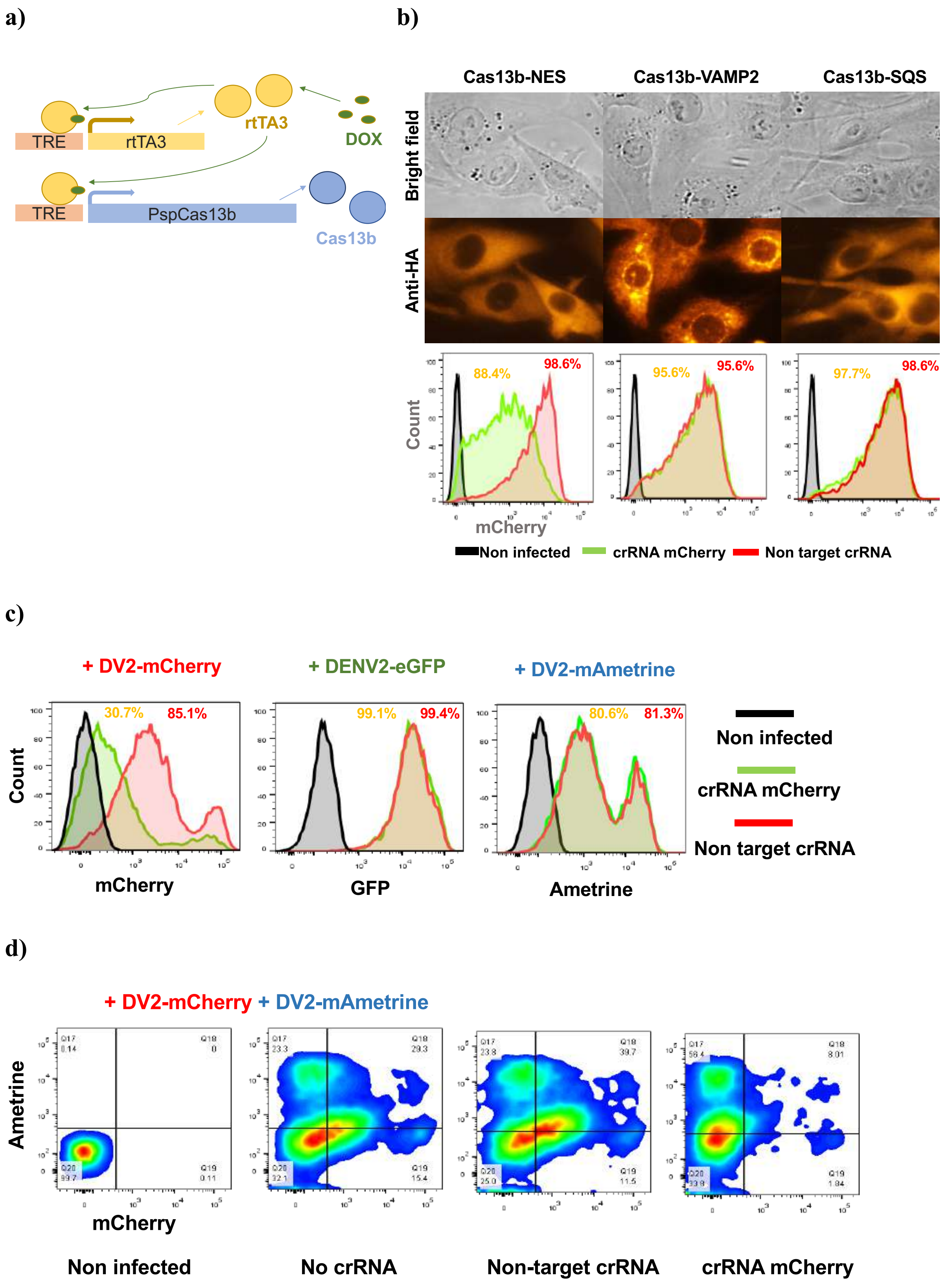

e)

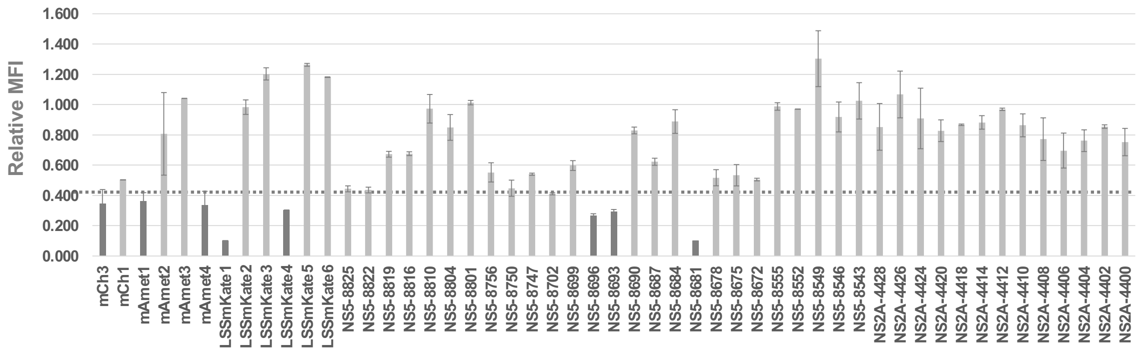

f)

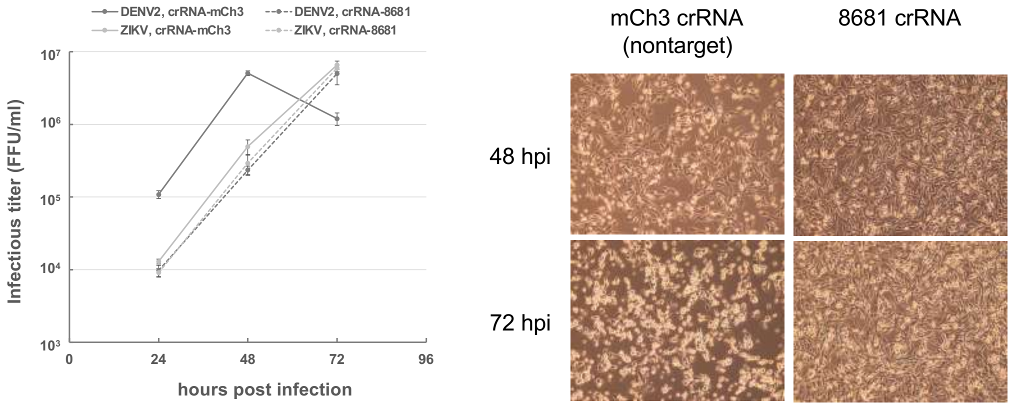

Supplementary Figure 1: Flaviviral suppression with CRISPR-Cas13b by inducible expression in BHK-21. a) Diagram describing the inducible expression system that controlled the expression of PspCas13b in BHK-21 cells. b) Cytoplasmic PspCas13b, but not ER-localized PspCas13b, could suppress DENV2-mCherry replication. The top panel is the bright-field images of BHK-21 expressing various forms of PspCas13b. The middle panel shows the same views of the top panel but with anti-HA signal representing the location of PspCas13b. The bottom panel is the histograms of mCherry intensity in DENV2-mCherry-infected BHK-21 with crRNAs and different forms of PspCas13b. The percentage of infection for each condition is color-coded in the histogram accordingly. c) Specific viral suppression of reporter DENV2 by an mCherry-targeting crRNA (mCh3 crRNA) in single-

virus infection setting. Viral suppression for each reporter DENV2 is shown as mean-fluorescent intensity (MFI) histograms from flow cytometry of the infected cells. The percentage of infection for each condition is color-coded in the histogram accordingly. d) Specific viral suppression of reporter DENV2 by mCh3 crRNA in co-infection setting. Viral suppression for each reporter DENV2 is represented as color density plots from flow cytometry of infected cells with different crRNAs. e) Summary bar plot of viral suppression activities of 51 crRNAs individually tested. Viral suppression is presented as a mean of relative MFI calculated from the ratio of MFI in BHK-21-Cas13b with experimental crRNA relative to the MFI in BHK-21-Cas13b with nontarget crRNA from duplicate measurements (error bar = standard deviation). Dark grey bars highlight the crRNA with relative MFI below 0.4. The details of the tested viruses and crRNA spacer sequences are listed in **Supplementary table 1**. f) Suppression of DENV2-16681 by a crRNA targeting DENV2 NS5 gene (8681 crRNA). The left plot compares replication kinetics of DENV2-16681 and ZIKV-SV0010/15 between BHK-21-Cas13b with 8681 crRNA and BHK-21-Cas13b with mCh3 crRNA (nontarget control) at 27-72 hpi (error bar = standard deviation). The measurements were done in triplicate. The right panel is representative bright-field images of the infected cells at 48 and 72 hpi.

a)

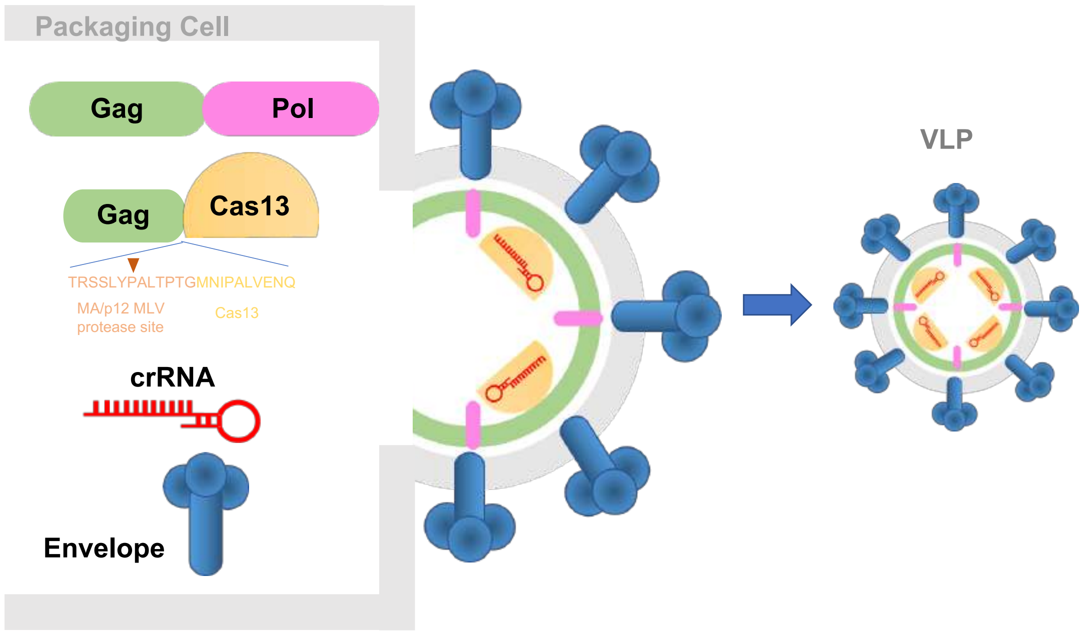
Packaging Cell
Gag
Pol
Gag
Cas13
TRSSLYPALTPTGMNIPALVENQ
MA/p12 MLV protease site
Cas13
crRNA
Envelope
VLP

b)

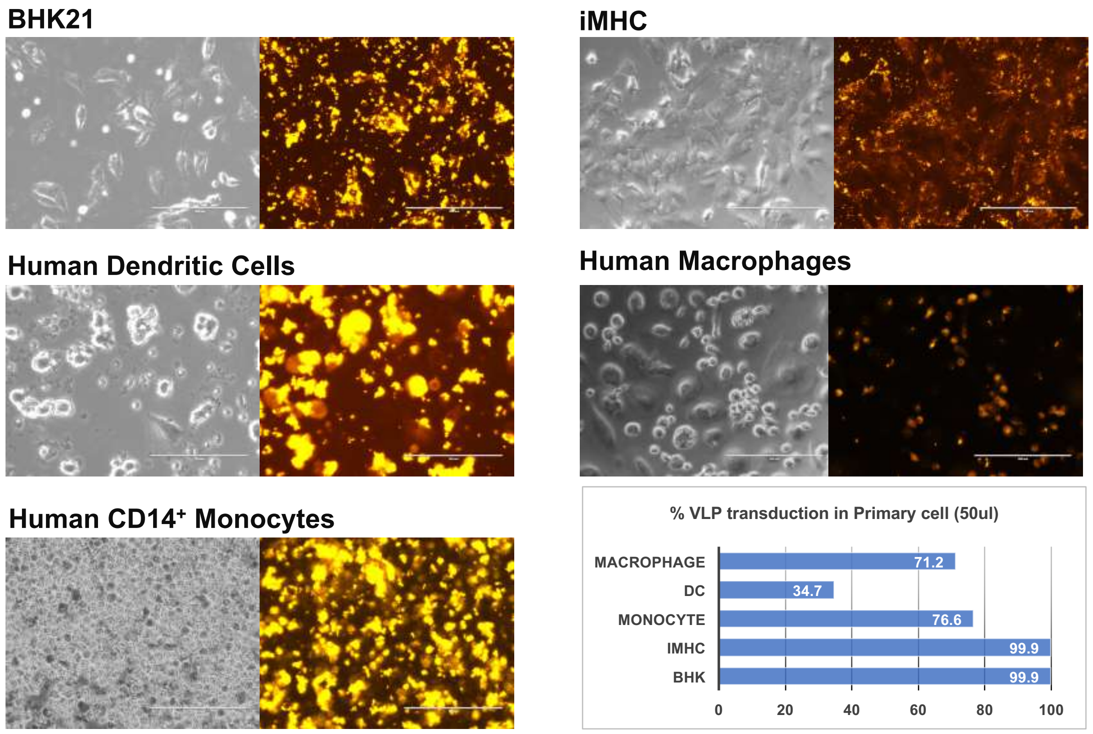
BHK21
iMHC
Human Dendritic Cells
Human Macrophages
Human CD14+ Monocytes
% VLP transduction in Primary cell (50ul)
MACROPHAGE
DC
MONOCYTE
IMHC
BHK
71.2
34.7
76.6
99.9
99.9
0
20
40
60
80
100

c)

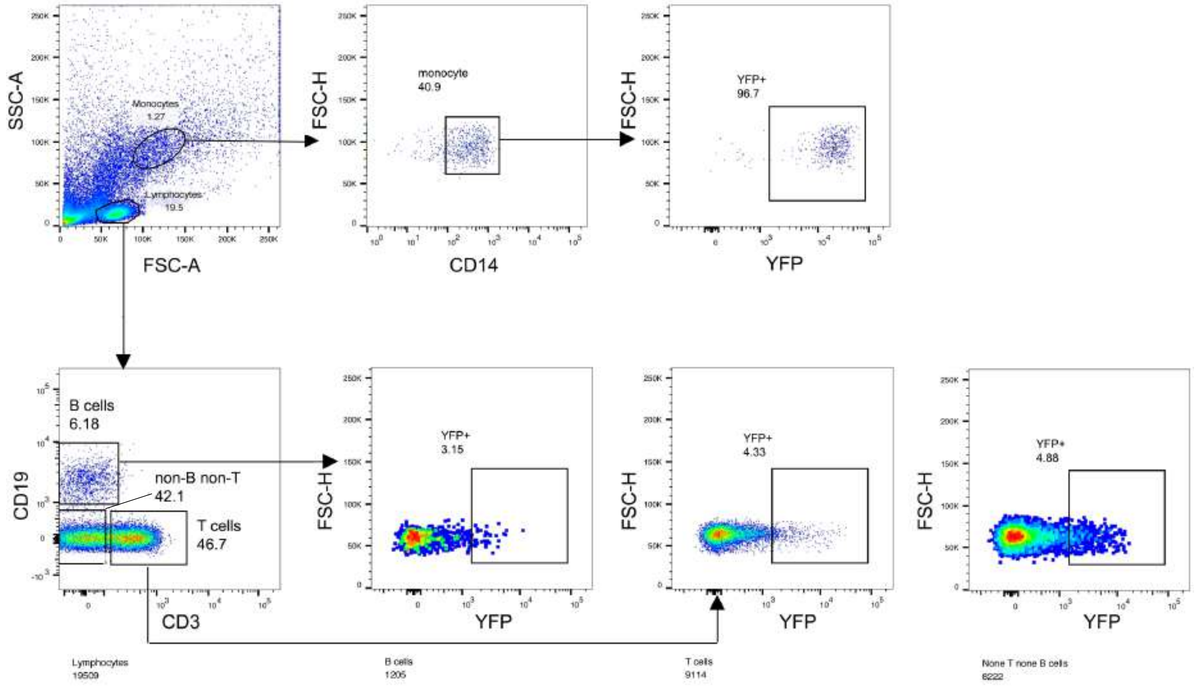

Supplementary Figure 2: VLP delivery of protein cargo into human primary target cells. a) Diagram showing the generation of VLP for PspCas13b RNP delivery (adapted from Mangeot et al., 2019). b) Delivery of YFP into various mammalian cells. The figure shows the results of transducing BHK-21, hDC, macrophages, iMHC, and CD14+ monocytes with VLP-YFP from fluorescent microscopy (bright-field images and fluorescent images) and percentage of transduction measured by flow cytometry. c) Flow cytometry of YFP delivery by VLP into different cell populations of human PBMC.

a)

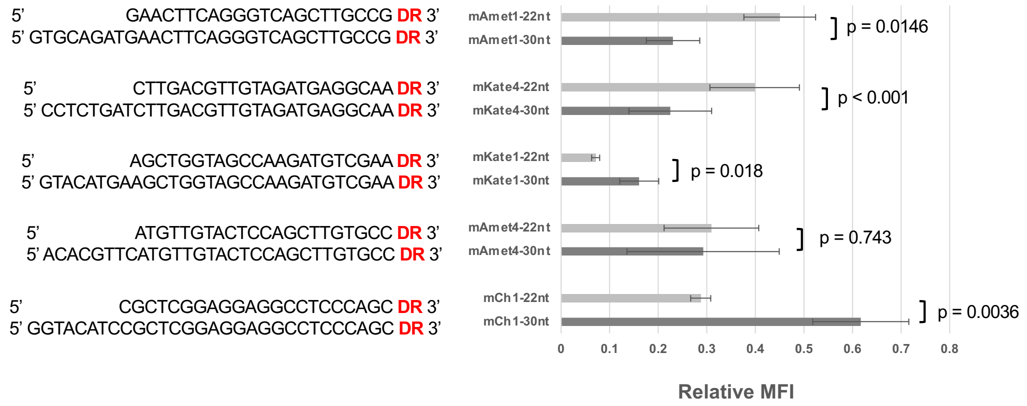

5' GAACTTCAGGGTCAGCTTGCCG DR 3'
5' GTGCAGATGAACTTCAGGGTCAGCTTGCCG DR 3'
5' CTTGACGTTGTAGATGAGGCAA DR 3'
5' CCTCTGATCTTGACGTTGTAGATGAGGCAA DR 3'
5' AGCTGGTAGCCAAGATGTCGAA DR 3'
5' GTACATGAAGCTGGTAGCCAAGATGTCGAA DR 3'
5' ATGTTGTACTCCAGCTTGTGCC DR 3'
5' ACACGTTCATGTTGTACTCCAGCTTGTGCC DR 3'
5' CGCTCGGAGGAGGCCTCCCAGC DR 3'
5' GGTACATCCGCTCGGAGGAGGCCTCCCAGC DR 3'
mAmet1-22nt
mAmet1-30nt
mKate4-22nt
mKate4-30nt
mKate1-22nt
mKate1-30nt
mAmet4-22nt
mAmet4-30nt
mCh1-22nt
mCh1-30nt
p = 0.0146
p < 0.001
p = 0.018
p = 0.743
p = 0.0036
0 0.1 0.2 0.3 0.4 0.5 0.6 0.7 0.8
Relative MFI

b)

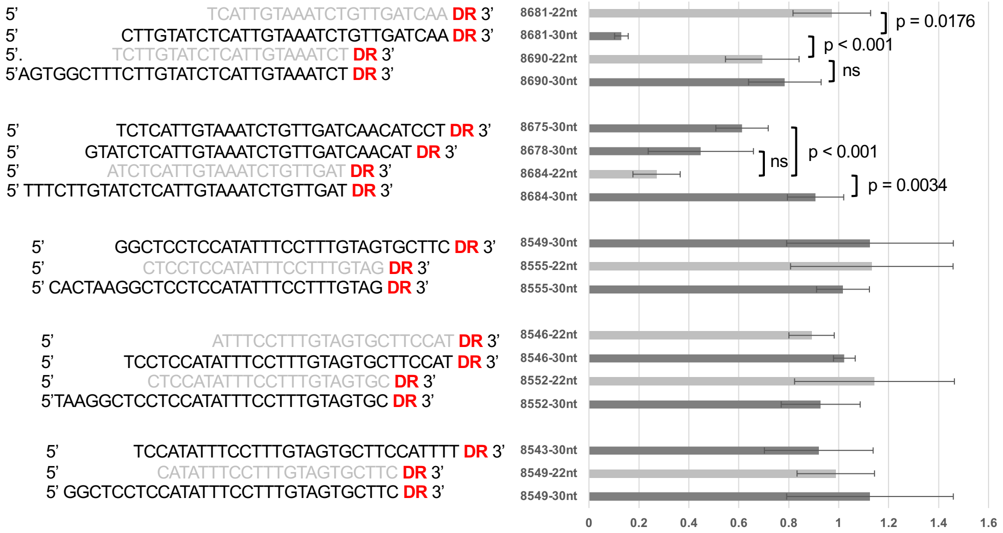

5' TCATTGTAAATCTGTTGATCAA DR 3'
5' CTTGTATCTCATTGTAAATCTGTTGATCAA DR 3'
5'. TCTTGTATCTCATTGTAAATCT DR 3'
5'AGTGGCTTTCTTGTATCTCATTGTAAATCT DR 3'
5' TCTCATTGTAAATCTGTTGATCAACATCCT DR 3'
5' GTATCTCATTGTAAATCTGTTGATCAACAT DR 3'
5' ATCTCATTGTAAATCTGTTGAT DR 3'
5' TTTCTTGTATCTCATTGTAAATCTGTTGAT DR 3'
5' GGCTCCTCCATATTTCCTTTGTAGTGCTTC DR 3'
5' CTCCTCCATATTTCCTTTGTAG DR 3'
5' CACTAAGGCTCCTCCATATTTCCTTTGTAG DR 3'
5' ATTTCCTTTGTAGTGCTTCCAT DR 3'
5' TCCTCCATATTTCCTTTGTAGTGCTTCCAT DR 3'
5' CTCCATATTTCCTTTGTAGTGC DR 3'
5'TAAGGCTCCTCCATATTTCCTTTGTAGTGC DR 3'
5' TCCATATTTCCTTTGTAGTGCTTCCATTTT DR 3'
5' CATATTTCCTTTGTAGTGCTTC DR 3'
5' GGCTCCTCCATATTTCCTTTGTAGTGCTTC DR 3'
8681-22nt
8681-30nt
8690-22nt
8690-30nt
8675-30nt
8678-30nt
8684-22nt
8684-30nt
8549-30nt
8555-22nt
8555-30nt
8546-22nt
8546-30nt
8552-22nt
8552-30nt
8543-30nt
8549-22nt
8549-30nt
p = 0.0176
p < 0.001
ns
ns
p < 0.001
p = 0.0034
0 0.2 0.4 0.6 0.8 1 1.2 1.4 1.6

c)

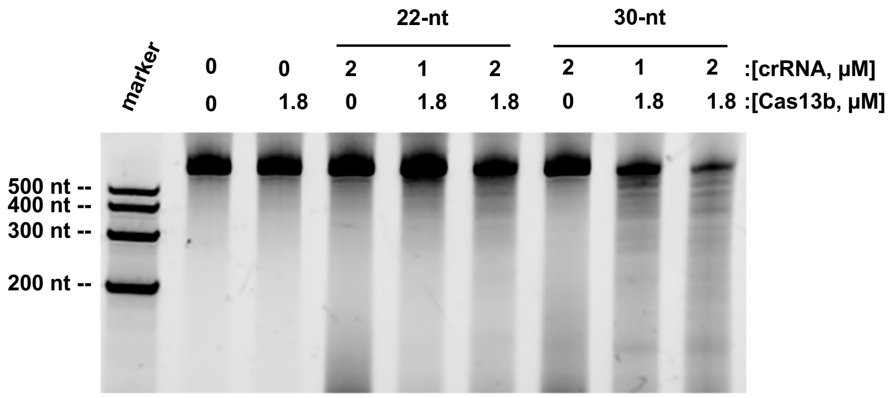

22-nt
30-nt
marker
0 0 2 1 2 2 1 2 :[crRNA, μM]
0 1.8 0 1.8 1.8 0 1.8 1.8 :[Cas13b, μM]
500 nt --
400 nt --
300 nt --
200 nt --

Supplementary Figure 3: The comparison of virus suppression activity between 22-nt and 30-nt crRNAs and in vitro cleavage. a) crRNA set for targeting fluorescent reporter genes. b) crRNA set for targeting NS5 gene of DENV2-16681. The measurements were done in four replicates. c) In vitro cleavage of mCherry RNA by PspCas13b + 22-nt mCh3 crRNA vs. 30-nt mCh3 crRNA.

Supplementary Table 1: The details and the knock-down activity of crRNAs shown in Supplementary Figure 1e. Knock-down ratio = relative MFI.

target	spacer sequence	test virus	knock-down ratio
mCh3	GGTACATCCGCTCGGAGGAGGCCTCCCAGC	DENV2-mCh	0.213
mCh1	TTGGCCTTGTAGGTGGTCTTGACCTCAGCG	DENV2-mCh	0.503
mAmet1	GTGCAGATGAACTTCAGGGTCAGCTTGCCG	DENV2-mAmet	0.432
mAmet2	CGCACGCTGAACTTGTGGCCGTTTACGTCG	DENV2-mAmet	0.938
mAmet3	TCAGCTCGATGCGGTTCACCAGGGTGTCGC	DENV2-mAmet	1.260
mAmet4	ACACGTTCATGTTGTACTCCAGCTTGTGCC	DENV2-mAmet	0.391
LSSmKate1	GTACATGAAGCTGGTAGCCAAGATGTCGAA	DENV2-mKate	0.085
LSSmKate2	TCGACCACCTTGATTCTCATGGTCTGGGTG	DENV2-mKate	0.837
LSSmKate3	AAGATGTCGAAGGCGAAGGGTAGAGGGCCG	DENV2-mKate	1.024
LSSmKate4	CCTCTGATCTTGACGTTGTAGATGAGGCAA	DENV2-mKate	0.258
LSSmKate5	CAGCCGAGTGTTTTCTTCTGCATCACAGGG	DENV2-mKate	1.075
LSSmKate6	CCAGTTTGCTAGGGAGGTCGCAGTATCTGG	DENV2-mKate	1.006
NS5-8825	TTGGTCATAGTGCCATGATGTTTCATGCTC	DENV2-mCh	0.553
NS5-8822	GTCATAGTGCCATGATGTTTCATGCTCTTG	DENV2-mCh	0.543
NS5-8819	ATAGTGCCATGATGTTTCATGCTCTTGCTT	DENV2-mCh	0.758
NS5-8816	GTGCCATGATGTTTCATGCTCTTGCTTTAT	DENV2-mCh	0.763
NS5-8810	TGATGTTTCATGCTCTTGCTTTATTTTTTC	DENV2-mCh	1.211
NS5-8804	TTCATGCTCTTGCTTTATTTTTTCTATTCT	DENV2-mCh	1.060
NS5-8801	ATGCTCTTGCTTTATTTTTTCTATTCTTTT	DENV2-mCh	1.263
NS5-8756	GTTTGGTATCTCACTTTCAATCCCGATGTT	DENV2-mCh	0.688
NS5-8750	TATCTCACTTTCAATCCCGATGTTACGGGT	DENV2-mCh	0.558
NS5-8747	CTCACTTTCAATCCCGATGTTACGGGTTCC	DENV2-mCh	0.610
NS5-8702	ATCCGGCTCGTAAGTGGCTTTCTTGTATCT	DENV2-mCh	0.463
NS5-8699	CGGCTCGTAAGTGGCTTTCTTGTATCTCAT	DENV2-mCh	0.674
NS5-8696	CTCGTAAGTGGCTTTCTTGTATCTCATTGT	DENV2-mCh	0.300
NS5-8693	GTAAGTGGCTTTCTTGTATCTCATTGTAAA	DENV2-mCh	0.331
NS5-8690	AGTGGCTTTCTTGTATCTCATTGTAAATCT	DENV2-mCh	1.034
NS5-8687	GGCTTTCTTGTATCTCATTGTAAATCTGTT	DENV2-mCh	0.703
NS5-8684	TTTCTTGTATCTCATTGTAAATCTGTTGAT	DENV2-mCh	1.108
NS5-8681	CTTGTATCTCATTGTAAATCTGTTGATCAA	DENV2-mCh	0.124
NS5-8678	GTATCTCATTGTAAATCTGTTGATCAACAT	DENV2-mCh	0.645
NS5-8675	TCTCATTGTAAATCTGTTGATCAACATCCT	DENV2-mCh	0.602
NS5-8672	CATTGTAAATCTGTTGATCAACATCCTTGA	DENV2-mCh	0.630
NS5-8555	CACTAAGGCTCCTCCATATTTCCTTTGTAG	DENV2-mCh	1.231
NS5-8552	TAAGGCTCCTCCATATTTCCTTTGTAGTGC	DENV2-mCh	1.209
NS5-8549	GGCTCCTCCATATTTCCTTTGTAGTGCTTC	DENV2-mCh	1.622
NS5-8546	TCCTCCATATTTCCTTTGTAGTGCTTCCAT	DENV2-mCh	1.143
NS5-8543	TCCATATTTCCTTTGTAGTGCTTCCATTTT	DENV2-mCh	1.275
NS2A-4428	CATCAGGTCGCCTTCCAAGGCGGAGATCGC	ZIKV	0.855
NS2A-4426	TCAGGTCGCCTTCCAAGGCGGAGATCGCAG	ZIKV	1.073
NS2A-4424	AGGTCGCCTTCCAAGGCGGAGATCGCAGTT	ZIKV	0.937
NS2A-4420	CGCCTTCCAAGGCGGAGATCGCAGTTTGCA	ZIKV	0.836
NS2A-4418	CCTTCCAAGGCGGAGATCGCAGTTTGCAGA	ZIKV	0.881
NS2A-4414	TTCCAAGGCGGAGATCGCAGTTTGCAGAAG	ZIKV	0.893
NS2A-4412	AAGGCGGAGATCGCAGTTTGCAGAAGACAC	ZIKV	0.985
NS2A-4410	GGCGGAGATCGCAGTTTGCAGAAGACACGA	ZIKV	0.882
NS2A-4408	CGGAGATCGCAGTTTGCAGAAGACACGAGG	ZIKV	0.794
NS2A-4406	GAGATCGCAGTTTGCAGAAGACACGAGGCC	ZIKV	0.699
NS2A-4404	GATCGCAGTTTGCAGAAGACACGAGGCCAA	ZIKV	0.779
NS2A-4402	TCGCAGTTTGCAGAAGACACGAGGCCAAGG	ZIKV	0.868
NS2A-4400	GCAGTTTGCAGAAGACACGAGGCCAAGGCC	ZIKV	0.758
